# Non-Renal Effects and the Risk Assessment of Environmental Cadmium Exposure

**DOI:** 10.1289/ehp.1307110

**Published:** 2014-02-25

**Authors:** Agneta Åkesson, Lars Barregard, Ingvar A. Bergdahl, Gunnar F. Nordberg, Monica Nordberg, Staffan Skerfving

**Affiliations:** 1 Institute of Environmental Medicine, Karolinska Institutet, Stockholm, Sweden; 2 Department of Occupational and Environmental Medicine, Sahlgrenska University Hospital and Academy, Gothenburg, Sweden; 3 Occupational and Environmental Medicine, Department of Public Health and Clinical Medicine, and; 4 Department of Biobank Research, Umeå University, Umeå, Sweden; 5 Division of Occupational and Environmental Medicine, Department of Laboratory Medicine, University Hospital, Lund, Sweden

## Abstract

Background: Exposure to cadmium (Cd) has long been recognized as a health hazard, both in industry and in general populations with high exposure. Under the currently prevailing health risk assessment, the relationship between urinary Cd (U-Cd) concentrations and tubular proteinuria is used. However, doubts have recently been raised regarding the justification of basing the risk assessment on this relationship at very low exposure.

Objectives: Our objective was to review available information on health effects of Cd exposure with respect to human health risk assessment.

Discussion: The associations between U-Cd and urinary proteins at very low exposure may not be due to Cd toxicity, and the clinical significance of slight proteinuria may also be limited. More importantly, other effects have been reported at very low Cd exposure. There is reason to challenge the basis of the existing health risk assessment for Cd. Our review of the literature found that exposure to low concentrations of Cd is associated with effects on bone, including increased risk of osteoporosis and fractures, and that this observation has implications for the health risk assessment of Cd. Other effects associated with Cd should also be considered, in particular cancer, although the information is still too limited for appropriate use in quantitative risk assessment.

Conclusion: Non-renal effects should be considered critical effects in the health risk assessment of Cd.

Citation: Åkesson A, Barregard L, Bergdahl IA, Nordberg GF, Nordberg M, Skerfving S. 2014. Non-renal effects and the risk assessment of environmental cadmium exposure. Environ Health Perspect 122:431–438; http://dx.doi.org/10.1289/ehp.1307110

## Introduction

Exposure to cadmium (Cd) has long been recognized as a health hazard, both in industry and in general populations with high exposure. There is widespread low-level Cd contamination of agricultural soil in many areas of the world. Because Cd is easily taken up by crops such as rice, wheat, vegetables, and potatoes and occurs in high concentrations in shellfish, offal, and certain seeds, the exposure to Cd from food in many areas is high enough to be of importance to human health [European Food Safety Authority (EFSA) 2009a; World Health Organization (WHO) 2011]. Additional concern stems from the fact that exposure may not be decreasing, except in some areas that were once highly contaminated. Tobacco smoking further increases Cd exposure.

The toxic effects of Cd were initially considered to be limited to lung and kidney damage (due to occupational exposure) and kidney damage, osteomalacia, and fractures (due to dietary exposure—“itai-itai disease”; reviewed by Nordberg et al. 2007). Until now, health risk assessment for both occupational exposure and long-term food-based exposure has been based on kidney effects, with tubular proteinuria considered to be the critical effect in humans. According to the U.S. Environmental Protection Agency (EPA) the critical effect is the first adverse effect, or its known precursor, that occurs in the most sensitive species as the dose rate of an agent increases (U.S. EPA 2014a). The dose–response assessment in the risk assessment process in humans relies on the relationship between Cd excreted in urine (urinary Cd, U-Cd) and urinary markers of early renal tubular effects (EFSA 2009a; WHO 2011). In recent years, however, other Cd-related adverse effects have been reported at low-level environmental exposures. We aimed to review the available information on those effects, to compare them with the kidney effects, and to indicate alternatives for risk assessors.

## Toxicokinetics of Cd

After uptake, Cd in blood plasma is bound to albumin and metallothionein (MT). Because of the small size of MT, Cd-MT is readily filtered through the glomeruli and reabsorbed by the proximal tubuli and thus accumulates in the kidney cortex, where a major part of the body burden is located (Nordberg et al. 2007). Because the half-life of Cd in the kidney is > 10 years (Akerström et al. 2013a; Amzal et al. 2009) and a strong association is observed between concentrations of Cd in the kidney and urine (Akerström et al. 2013a), the biomarker U-Cd reflects lifelong kidney accumulation, which in turn mirrors the long-term total body burden. The majority of circulating Cd is bound in erythrocytes. Blood cadmium (B-Cd) is another possible biomarker of exposure. Because of the shorter half-life of Cd in blood, B-Cd reflects changes in exposure more closely than U-Cd (Liang et al. 2012). Numerous factors such as age, smoking status, and gastrointestinal Cd absorption [e.g., low iron stores increase the gastrointestinal absorption of Cd (Åkesson et al. 2002; Berglund et al. 1994)] influence the relationship between dietary Cd exposure and U-Cd. In the Supplemental Material, Figure S1 shows the predicted relationship between estimated average long-term dietary Cd intake and the corresponding U-Cd concentration as modeled for 50-year-old women with a constant daily Cd intake (Amzal et al. 2009).

## Toxic Effects of Cd on Kidneys

*Renal tubular dysfunction.* Proteinuria is well-established as an adverse effect of Cd exposure. Long-term exposure resulting in U-Cd > 4 μg/g creatinine (cr) and/or B-Cd > 4 μg/L impairs renal tubular reabsorptive function, as shown by increased urinary excretion of low-molecular weight proteins (LMWP) such as β_2_-microglobulin (B2M), α_1_-microglobulin (A1M), and retinol-binding protein (EFSA 2009a; Järup and Åkesson 2009; Nordberg et al. 2007). The use of these LMWP as markers of adverse effect is supported by long-term follow-up surveys in Japan, where populations with Cd-induced tubular dysfunction demonstrated increased mortality due to renal, cardiovascular, and cerebrovascular disorders, particularly when B2M exceeded 1,000 μg/g cr (Nishijo et al. 2006).

The EFSA (2009b) summarized the available data in a meta-analysis in order to establish a dose–response relationship between U-Cd and B2M excretion. Both EFSA and the Joint Food and Agriculture Organization of the United Nations (FAO)/WHO Expert Committee on Food Additives modeled the relationships in their risk assessments (EFSA 2009a; WHO 2011), and both used 4–5 μg Cd/g cr as the point at which an increase in urinary B2M (U-B2M) was considered to occur, but arrived at different tolerable intakes. A weakness of both risk assessments is the fact that several studies of high quality were excluded from the meta-analysis—either because they used 24-hr urine sampling (instead of expressing the excretion per gram of creatinine in spot samples) or because they did not use U-B2M at all because of its susceptibility to breakdown at low urinary pH levels.

Several studies of B2M (Chen et al. 2006b; Hong et al. 2004; Olsson et al. 2002) and other LWMP (Åkesson et al. 2005; de Burbure et al. 2006; Järup et al. 2000) have reported positive associations between U-Cd and protein excretion at U-Cd < 4–5 μg/g cr, and even at U-Cd as low as < 1 μg/g cr. One study also reported an association between LMWP and B-Cd < 1 μg/L (Åkesson et al. 2005). Whether these associations represent a causal relationship is discussed in the section “Low-level urinary Cd, proteinuria and causality.”

*Glomerular dysfunction.* Some studies have reported associations between low-level Cd exposure and estimated glomerular function in cross-sectional analyses. In 700 elderly women estimated glomerular filtration rate (eGFR), based on serum cystatin C or serum creatinine, was statistically significantly lower at U-Cd 0.75–1.0 μg/g cr than at U-Cd < 0.5 μg/g cr (Åkesson et al. 2005; Suwazono et al. 2006). Moreover, eGFR was decreased at B-Cd > 1 μg/L compared with B-Cd < 0.5 μg/L (Åkesson et al. 2005). Although the associations became nonsignificant in the relatively small subgroup of never-smokers, a trend still appeared. Navas-Acien et al. (2009) analyzed B-Cd data from > 14,000 individuals in the United States and found lower odds of low eGFR at B-Cd > 0.6 μg/L (median 1 μg/L) compared with < 0.2 μg/L [odds ratio (OR): 1.32; 95% CI: 1.04, 1.68], although no associations were observed for the much smaller subgroup for whom U-Cd data were available (Ferraro et al. 2010). The increased OR for B-Cd > 0.6 μg/L was not present among never-smokers (Navas-Acien et al. 2009). An association between B-Cd and low eGFR was also found among Korean women but not men (Hwangbo et al. 2011; Myong et al. 2012). Estimates of GFR from creatinine or cystatin C in blood have been shown to be imprecise and biased when GFR is normal or near normal (Issa et al. 2008; Murata et al. 2011; Tent et al. 2010). Therefore, even if the studies on eGFR suggest associations with Cd concentrations, they do not provide definitive evidence of clinically relevant reduced GFR at low-level Cd exposures.

Apart from a change in the GFR, proteinuria is the hallmark of glomerular disease, and initially albumin excretion increases most. Albumin has a molecular size of about 65 kDa, which is above the threshold in the glomerular basement membrane barrier. Thus, elevated albumin in urine indicates damage of the integrity of the barrier. Several studies have demonstrated increased excretion of urinary albumin (U-Alb) in Cd-exposed workers and populations (Bernard et al. 1979; Buchet et al. 1980; Chen et al. 2006a; Ferraro et al. 2010; Jin et al. 2004; Liang et al. 2012; Navas-Acien et al. 2009). In an environmentally exposed Chinese population, elevated U-Alb concentrations were reversed over the 8-year period after cessation of the consumption of Cd-contaminated rice and subsequent lower dietary Cd exposure, but changes in the excretion of LMWP were not reversible (Liang et al. 2012).

*Renal failure.* Several studies of high Cd exposure have shown associations between U-Cd and mortality from renal diseases (Nakagawa et al. 2006; Nishijo et al. 2006). An increased risk of end-stage renal disease (ESRD) was found in an ecological Swedish study that combined occupationally and environmentally exposed subjects residing in areas near battery plants (Hellström et al. 2001). On the other hand, an ecological study in Japan showed no association between mortality associated with renal failure and Cd concentrations in local brown rice (Koizumi et al. 2010). Only one study of prospective design has been published on ESRD in relation to low-level Cd biomarkers (Sommar et al. 2013), and this case–referent study did not find Cd concentrations in erythrocytes at baseline to be a statistically significant risk factor for ESRD after adjusting for potential confounders.

*Low-level U-Cd, proteinuria, and causality.* Bernard (2008) proposed that the associations observed between very low-level U-Cd and proteinuria may not be caused by Cd toxicity. Alternative explanations are that the associations are confounded by smoking or co-excretion of Cd and proteins due to variation in renal physiology, as discussed below.

Tobacco smoking substantially increases Cd exposure and, thereby, both B-Cd and U-Cd. If smoking also causes proteinuria (Haddam et al. 2011) independently of the Cd content in smoke, then it is an important confounder. In occupationally exposed workers, a weaker positive association between LMWP and U-Cd has been observed in never-smokers as compared with smokers (Chaumont et al. 2011; Haddam et al. 2011).

There are physiological mechanisms that could result in an association between excretion of Cd and LMWP without Cd toxicity being the cause. After filtration through the glomeruli, LMWP competes with albumin (in small amounts) and Cd-MT for reabsorption in the proximal tubule. LMWP and Cd-MT seem to have similar affinity for tubular binding sites (Bernard 2008; Chaumont et al. 2012), and so normal physiological changes in renal tubular reabsorption function can result in the co-excretion of Cd and LMWP. Varying diuresis (urinary flow rate) is an example of such normal renal physiological variability (Akerström et al. 2013b). This mechanism could be the reason for associations between excretion of Cd and LMWP among healthy teenagers with very low U-Cd (Chaumont et al. 2012). Akerström et al. (2013b) reported a positive association between the excretion of Cd, A1M, and albumin within individuals with very low U-Cd (< 1 μg/g cr) irrespective of adjustment for variation in dilution; moreover, urine flow rate had a positive impact on the excretion of Cd. Thus, it is possible that normal physiological variability in renal reabsorption of LMWP causes the increase in U-Cd by inhibiting tubular uptake of MT-bound Cd; in other words, this is a possible case of reverse causality (Chaumont et al. 2012).

Although there is no reason to question the effect of Cd exposure on renal tubules at high exposure (U-Cd > 4 μg/g cr), the associations observed at low levels could be influenced by the factors mentioned above. Other factors should also be considered, such as the ability to synthesize MT and the occurrence of MT antibodies (Nordberg et al. 2012). Thus, although a toxic effect cannot be ruled out at exposures corresponding to U-Cd < 1 μgCd/g cr (values that generally occur among nonsmokers in many populations worldwide), normal physiology is likely to be an important determinant (Akerström et al. 2013b; Chaumont et al. 2012). This makes it difficult to interpret any associations.

The effects of renal physiology are most likely eliminated when B-Cd, instead of U-Cd, is used as a marker of Cd exposure in relation to kidney effects markers in urine. Studies using B-Cd and LMWP in never-smokers would shed light on this issue, but such studies are demanding regarding population size and analytical performance. One study observed a statistically significant association in never-smokers between B-Cd and LMWP excretion (U-A1M) within the normal range (Åkesson et al. 2005). A U-Cd concentration of 1 μg/g cr corresponds to a B-Cd concentration of about 1 μg/L, although the variation is wide.

Although long-term Cd-induced tubular proteinuria (e.g., high U-B2M) may be a risk factor for renal failure and mortality (Nishijo et al. 2006), the public health impact of Cd-related increases in biomarkers of tubular dysfunction within the normal range is unknown.

## Toxic Effects of Cd on Bone

It has long been well-established that excessive exposure to Cd may cause itai-itai disease, which occurs after manifestation of kidney damage and leads to osteomalacia and/or osteoporosis with multiple fractures (Nordberg et al. 2007).

A long series of recent cross-sectional and prospective studies of different populations, mainly from Belgium, Sweden, the United States, and China—some of them very large—clearly demonstrate associations between Cd exposure and low bone mineral density, as well as an increased risk of osteoporosis (Table 1). Most of these studies used dual-energy X-ray absorptiometry and defined low bone mass/osteoporosis based on the *z*-score or *T*-score.

**Table 1:** Studies of the relationship between Cd exposure and bone effects.

Country; study population; sex	Age; no. of participants	Study design; bone effect measure	Threshold of bone effect	Smoking adjustment or stratification	Reference
Studies with bone mineral density or osteoporosis as outcome
Belgium; general population; men and women	Mean, 44 years; *n *= 506	Prospective; density	Association with U-Cd (mean, ~ 1.0 μg/g cr) in women; no threshold	No effect of smoking	Staessen et al. 1999
South Sweden; general population and battery workers; women and men	Means, 41 and 44 years; *n *= 1,064	Cross-sectional; osteoporosis (*z*-score ≥ 1)	10% excess risk at U-Cd 0.5–3.0 μg/g cr, vs. < 0.5 μg/g cr	Adjusted	Alfvén et al. 2000
Japan; general population; women	Range, 40–86 years; *n *= 908	Cross-sectional; density (ultrasound; calcaneus stiffness)	Density negatively correlated with U-Cd (mean, 2.9 μg/g cr)	No adjustment or stratification	Honda et al. 2003
China; general population in areas with varying contamination of rice; women and men	Means, 50 and 55 years; *n *= 790	Cross-sectional with longitudinal components; density and osteoporosis (*T*-score ≥ 2.5)	Effects at mean U-Cd 2.3–13 μg/g cr; no observed reversibility (Chen et al. 2009)	Adjusted	Chen et al. 2009; Jin et al. 2004; Wang et al. 2003
Sweden; fishermen and their wives	Medians, 59 and 62 years; *n *= 380	Cross-sectional; density and biochemical markers	No association with U-Cd (medians, 0.22, 0.34 μg/g cr)	Adjusted	Wallin et al. 2005
Japan; farmers from areas with varying contamination of rice; women	Range, 41–75 years; *n *= 1,243	Cross-sectional; density (< 80% of young adults) and biochemical markers	No effect of U-Cd (< ~ 0.3–27 μg/g cr)	Never-smokers only	Horiguchi et al. 2005
South Sweden; general population; women	Range, 53–64 years; *n *= 820	Cross-sectional; density BMDL5/BMDL10 (5%/10% additional risk) and biochemical markers	BMDL5: U-Cd 1.0 μg/g cr; BMDL10: U-Cd 1.6 μg/g cr	Stratified; association also among never-smokers	Åkesson et al. 2006; Suwazono et al. 2010
United States, NHANES; general population; women	≥ 50 years; *n *= 3,311	Cross-sectional; osteoporosis of the hip (*T*-score ≥ 2.5)	U-Cd 0.5–1.0 μg/g cr gave a 43% increased risk	Stratified; borderline significance among never-smokers only	Gallagher et al. 2008
Belgium; general population; women	Mean, 49 years; *n *= 294	Cross-sectional; density and biochemical markers	Negative association between U-Cd and BMD in menopause (U-Cd ≥ ~ 1.3 μg/g cr)	Adjusted	Schutte et al. 2008
Poland; general population in Cd-polluted areas; women and men	Range, 18–76 years; *n *= 270	Cross-sectional; density (*T*-score) and biochemical markers	No association after adjustments (GM U-Cd was 1.1 μg/g cr in women and 0.9 μg/g cr in men)	Adjusted	Trzcinka-Ochocka et al. 2009
South Sweden; general population; women	Range, 60–70 years; *n *= 908	Cross-sectional; density and biochemical markers	Association with B-Cd (median, ~ 0.4 μg/L)	No association in smoking-adjusted model	Rignell-Hydbom et al. 2009
United States, NHANES; general population; women and men	Range, 30–90 years; *n *= 10,978	Cross-sectional; osteoporosis of the hip (*T*-score ≥ 2.5)	U-Cd 1.0–2.0 μg/g cr gave a 78% increased risk	Adjusted	Wu et al. 2010
Belgium; workers; men	Range, 24–64 years; *n *= 83	Cross-sectional; osteoporosis (*T*-score ≥ 2.5)	U-Cd > 1.9 μg/g cr gave a 10-fold increased risk	Adjusted	Nawrot et al. 2010
Sweden; general population; women	Range, 56–69 years; *n *= 2,688	Cross-sectional; density, total body osteoporosis hip and spine (*T*-score ≥ 2.5)	U-Cd 0.50–0.75 and > 0.75 vs. U-Cd < 0.5 μg/g (referent); OR = 1.61 (1.20–2.16) and 1.95 (1.30–2.93), respectively; in never-smokers, OR, 1.27 (0.75–2.14) and 4.24 (1.99–9.04), respectively	Stratified; associations in never-smokers	Engström et al. 2011
Sweden; general population; women	Range, 56–69 years; *n *= 2,676	Prospective; density, total body osteoporosis hip and spine (*T*-score ≥ 2.5)	OR = 1.32 (95% CI: 1.02–1.71) for dietary Cd > median (13 μg/day) vs. lower; combined high dietary and U-Cd (> 0.5 μg/g cr) gave OR = 2.49 (95% CI: 1.71–3.63) among all women and 2.65 (95% CI: 1.43–4.91) among never-smokers	Adjusted; associations in never-smokers	Engström et al. 2012
Studies with fractures as outcome
Belgium; general population; women and men	Mean, 44 years; *n *= 506	Prospective; any fracture	Mean U-Cd, 1.0 μg/g cr; RR = 1.7 (95% CI: 1.18–2.57) for wrist fracture at a 2–fold increase of U-Cd in women, not in men; no threshold reported	No effect of smoking	Staessen et al. 1999
China; general population in areas with varying Cd-contamination of rice; women and men	Means, 50 and 55 years; *n *= 790	Retrospective; collection of low-impact fractures	Mean U-Cd, 9.2–13, vs. 1.6–1.8 μg/g cr caused age-standardized RR 4.1 (95% CI: 1.55–6.61) in men and 2.5 (95% CI: 1.42–3.54) in women	No	Wang et al. 2003
South Sweden; general population and workers; women and men	Range, 16–81 years; *n *= 1,021	Retrospective; forearm fractures	HR = 3.5 (95% CI: 1.1–11) at U-Cd 2–4 μg/g cr vs. < 0.5 μg/g cr	Adjusted	Alfvén et al. 2004
Sweden; general population; women	Range, 56–69 years; *n *= 2,688	Both prospective and retrospective components; any first fracture, first osteoporotic fracture, first distal forearm fracture	OR = 2.0–2.2 comparing U-Cd > 0.50 μg/g cr with lower concentrations in never-smokers; corresponding OR for all women 1.15–1.29 (non­significant)	Stratified; associations were only statistically significant in never-smokers	Engström et al. 2011
Sweden; general population; women	Range, 56–69 years; *n *= 2,676	Prospective for dietary Cd and combined prospective and retrospective for U-Cd; any first fracture	OR = 1.31 (95% CI: 1.02–1.69) for dietary Cd > median (13 μg/day) vs. ≤ median; corresponding OR in never-smokers 1.54 (95% CI: 1.06–2.24); combined high dietary and U-Cd (> 0.5 μg/g cr) OR = 1.46 (95% CI: 1.00–2.15) among all women, and 3.05 (95% CI: 1.66–5.59) among never-smokers	Stratified; slightly higher OR in never-smokers	Engström et al. 2012
Sweden; general population; men	Range, 45–79 years; *n *= 22,173	Prospective; any first fracture, hip fractures	HR = 1.2 comparing highest with lowest Cd intake tertiles	Stratified; association for hip fracture also among never-smokers only	Thomas et al. 2011
Abbreviations: BMDL, benchmark dose lower confidence bound; density, bone mineral density; GM, geometric mean; HR, hazard ratio; ND, not done; NHANES, National Health and Nutrition Examination Survey; RR, relative risk. Standardized scores represent the number of SDs of density below the average in a population of young adults (*T*-score) or a population of similar age (*z*-score).					

The relationship between osteoporosis and fracture risk is well-established (Mackey et al. 2007; Marshall et al. 1996); osteoporosis at a skeletal site is highly predictive of a fracture in the same area. The Cd-associated increased risk of osteoporosis observed in some studies is thus of concern (Alfvén et al. 2000; Engström et al. 2011, 2012; Gallagher et al. 2008; Nawrot et al. 2010; Staessen et al. 1999; Wang et al. 2003; Wu et al. 2010) (Table 1).

Most bone studies have used U-Cd to explore associations (Table 1). Although these associations were present at very low-exposure levels, it is not likely that they represent reverse causation, that is, that the bone effects cause the increased U-Cd (e.g., that bone-derived proteins bind Cd and are excreted into urine). In addition to the studies based on biomonitoring of exposure, two were based on dietary Cd exposure, combining individual food consumption data from a food-frequency questionnaire with data on Cd content in food (Engström et al. 2012; Thomas et al. 2011). Both Engström et al. (2012) and Thomas et al. (2011) observed associations with osteoporosis and/or fracture incidence, even though the exposure misclassification is likely to be larger than for the biomarkers with this method. Decreased bone mineral density with increasing B-Cd has been described in a few studies. In Alfvén et al. (2002), B-Cd was < 1 μg/L (corresponding to an average U-Cd < 1 μg/g cr), but the study population included subjects who had previously had higher Cd exposure. In a study by Nordberg et al. (2002), the exposure levels were very high (> 20 μg/L). Nevertheless, the fact that associations between Cd and effects on bone were observed by the use of three different exposure assessment methods (urine, blood, and dietary intake) reduces the likelihood that the results were due to confounding.

Another aspect in the interpretation of the studies on bone effects is the potential confounding by smoking (Law and Hackshaw 1997; Ward and Klesges 2001). Because tobacco smoke may well contain other agents that affect bone mineral density and fracture risk, such potential confounding must be considered to understand the actual association between Cd exposure and risk. In addition, smoking cessation is associated with a beneficial effect on bone (Oncken et al. 2006), whereas U-Cd concentrations remain essentially unchanged after smoking cessation. A few studies did stratify by smoking status, and significant (Åkesson et al. 2006; Engström et al. 2011, 2012; Thomas et al. 2011) or close to significant associations (Gallagher et al. 2008) were observed between Cd exposure and bone effect in never-smokers (Table 1). Indeed, two studies, based on dietary Cd intake rather than U-Cd, even reported stronger association in never-smokers than in all participants/ever-smokers (Engström et al. 2012; Thomas et al. 2011). This strongly supports the likelihood of an association with Cd that is independent of tobacco smoke.

Four studies failed to establish any statistically significant Cd-related effect on bone mineral density (Table 1). These null findings may be partly due to very low and/or narrow distribution of exposure, limited statistical power, and perhaps too young an age among the study populations. A small study of 380 men and women, 49–77 years of age, with low exposure showed no significant association between U-Cd and bone mineral density (Wallin et al. 2005). Another small study (170 women and 100 men, 18–79 years of age) from Poland showed significant associations between U-Cd and B-Cd, on the one hand, and markers of bone mineral density on the other; however, the association became nonsignificant after adjustment (Trzcinka-Ochocka et al. 2009). The relatively young age of the participants may have contributed to the lack of significant associations. Horiguchi et al. (2005) did not observe any association between U-Cd or B-Cd and bone mineral density in 1,243 women consuming rice with varying amounts of Cd contamination. However, because the statistical model could have resulted in overadjustment, the results were not conclusive. Finally, a study of 908 Swedish women found that bone mineral density and markers of bone metabolism were statistically significantly associated at low B-Cd (Rignell-Hydbom et al. 2009). However, after adjusting for smoking, there was no significant correlation, and the statistical power was too low for a meaningful exclusive analysis of the never-smokers. Therefore, we considered these four studies to be inconclusive.

The levels of Cd exposure associated with decreased bone mineral density and increased risk of osteoporosis and fractures vary (Table 1). Cross-sectional and prospective studies reported associations at U-Cd 0.5–2 μg/g cr.

The mechanisms of bone effects considered secondary to kidney damage include deficient reabsorption of calcium in the renal tubuli and compromised activation of vitamin D in the renal cortex. Several of the studies of bone effects also assessed kidney effects in parallel. The current understanding is that kidney effects are important in high Cd exposure situations (Jin et al. 2004), and the osteoporosis that is observed at low Cd exposure may be independent of kidney effects (Åkesson et al. 2006; Nawrot et al. 2010; Schutte et al. 2008). In accordance with this, there was no association between circulating concentrations of the active metabolite of vitamin D and U-Cd or markers of bone metabolism in women with relatively low Cd exposure despite significant associations between U-Cd and bone mineral density and bone metabolic markers (Engström et al. 2009).

There is growing evidence that Cd has a direct toxic effect on bone. Cd accumulates in osteocytes, the periosteum, and bone marrow but not in the hydroxyapatite (Lindh et al. 1981). Experimental studies demonstrate skeletal effects of Cd *in vitro*, as well as *in vivo* in animals displaying no nephrotoxicity (Bhattacharyya 2009; Nordberg et al. 2007). Osteoclasts in culture are particularly sensitive to low Cd concentrations (Bhattacharyya 2009). In accordance with this, cross-sectional investigations have found a positive association between U-Cd and markers of bone resorption (Åkesson et al. 2006; Schutte et al. 2008) (Table 1), even in children (Sughis et al. 2011). As a consequence of increased release of calcium from bone to the circulation, the excess is excreted into urine. Because U-Cd was inversely associated with levels of parathyroid hormone (Åkesson et al. 2006; Schutte et al. 2008), the Cd-associated calciuria is most likely a result of increased bone resorption, rather than decreased tubular reabsorption, which would instead have resulted in a compensatory increase in parathyroid hormone.

The effect of Cd on the skeleton has been reported to be irreversible upon cessation of exposure. A longitudinal study from contaminated areas in China examined individuals living in areas with moderate (0.51 mg/kg) and heavy (3.7 mg/kg) exposure after their cessation of consuming Cd-polluted rice (Chen et al. 2009). The decrease in wrist bone mineral density in women over a period of 8 years was larger when baseline U-Cd and B-Cd were high compared with low-exposure groups.

In conclusion, the data point toward a direct effect of Cd on bone. Even in the absence of Cd-induced renal tubular dysfunction, low-level environmental exposure to Cd seems to mobilize bone minerals from the skeletal tissue. Effects on bone mineral density, osteoporosis, and increased fracture risk are reported to occur at U-Cd as low as 0.5–2 μg/g cr (Åkesson et al. 2006; Alfvén et al. 2000, 2004; Engström et al. 2011; Gallagher et al. 2008; Nawrot et al. 2010; Schutte et al. 2008; Staessen et al. 1999; Wu et al. 2010). Similar associations have been observed at corresponding dietary intake levels (Engström et al. 2012; Thomas et al. 2011). Such associations were also observed in studies where tobacco smoking could not be the cause (Åkesson et al. 2006; Engström et al. 2011, 2012; Thomas et al. 2011). The bone effects at high exposures do not appear to be reversible (Chen et al. 2009).

Fragility fractures represent a considerable public health problem, causing suffering as well as a burden to the individual and the society (Ström et al. 2011). Hence, on the individual and the population level, fractures are much more severe health outcomes than are the decrease of bone mineral density and increase of subclinical osteoporosis. The population attributable risk of dietary Cd for osteoporotic fractures was estimated to be about 7% and 13% in women and men, respectively (Engström et al. 2012; Thomas et al. 2011) in Sweden, where the exposure to Cd is at the low end in a global perspective (Hruba et al. 2012; Pawlas et al. 2013; Wennberg et al. 2006).

## Cancer

In their most recent evaluation, the International Agency for Research on Cancer (2012) reconfirmed that there is sufficient evidence of Cd being a human carcinogen, a conclusion based mainly on lung cancer studies of workers.

Studies of Cd exposure and cancer in the general population have found positive associations. In a Belgian prospective cohort of 994 persons, Nawrot et al. (2006) found that 24-hr U-Cd was associated with an increased risk of lung cancer [relative risk (RR) = 1.70 (95% CI: 1.13, 2.57) for a doubling of U-Cd (median 1.1 μg/24 hr)]; the study was simultaneously adjusted for, among other things, smoking and arsenic exposure. The risk was also increased in a geographical area with high Cd pollution, compared with one with low Cd pollution, and in relation to the Cd concentrations in soil (although confounding by arsenic exposure cannot be ruled out). In a Belgian case–control study of bladder cancer, Kellen et al. (2007) found an increased risk even after adjusting for smoking (OR = 5.7; 95% CI: 3.3, 9.9) in study subjects with high B-Cd (> 1 μg/L) compared with those with low B-Cd (< 0.2 μg/L).

Several mechanisms have been proposed for Cd-induced carcinogenicity, including aberrant gene expression, oxidative stress, inhibition of DNA damage repair (Jin et al. 2003), apoptosis (Joseph 2009), and epigenetic alterations (Arita and Costa 2009). A factor of particular interest is that Cd may mimic the *in vivo* effects of estrogen in reproductive tissues (Ali et al. 2010, 2012; Byrne et al. 2009; Johnson et al. 2003). Present evidence does not allow a quantification of estrogenic risks (Kortenkamp 2011), but hormone-related cancers may still be of special concern.

In two very large population-based cohorts of Swedish men or postmenopausal women with an estimated average dietary Cd intake of 19 μg/day for men and 15 μg/day for women (1.7 μg/kg and 1.6 μg/kg BW per week, respectively), statistically significantly increased incidences of endometrial (RR = 1.39; 95% CI: 1.04, 1.86), breast (RR = 1.21; 95% CI: 1.07, 1.36), and prostate (RR = 1.13; CI: 1.03, 1.24) cancer (but not ovarian cancer) were observed in study subjects in the highest tertiles of Cd exposure (Åkesson et al. 2008; Julin et al. 2011, 2012a, 2012b). Among never-smoking, non-overweight women without postmenopausal hormonal use, those who had a Cd intake above the median on two occasions 10 years apart had an RR of 2.9 (95% CI: 1.05, 7.79) for endometrial cancer (Åkesson et al. 2008). The median U-Cd concentration in these never smoking women (1,225 women) was 0.29 μg/g cr (5th–95th percentiles, 0.15–0.79 μg/g cr; Engström et al. 2011). In contrast, estimated dietary Cd exposure was not associated with the incidence of either total cancers or specific cancers in 90,000 Japanese men and women (Sawada et al. 2012), or with the incidence of postmenopausal breast cancer in 30,000 U.S. women (Adams et al. 2012a). However, the National Health and Nutrition Examination Survey prospectively showed that uterine and total cancer mortality were associated with increasing U-Cd (Adams et al. 2012b). Four case–control studies have been performed on breast cancer, all showing statistically significant associations with U-Cd (Gallagher et al. 2010; McElroy et al. 2006; Nagata et al. 2013). In a study including 246 breast cancer cases, McElroy et al. (2006) estimated a multivariable-adjusted OR of 2.29 (95% CI: 1.3, 4.2), comparing the highest quartile of U-Cd (> 0.58 μg/g cr) with the lowest (< 0.26 μg/g cr). Based on 153 breast cancer cases, Nagata et al. (2013) estimated an OR of 6.05 (95% CI: 2.90, 12.62) comparing the highest tertile of U-Cd (> 2.6 μg/g) with the lowest (< 1.7 μg/g). Similar results were observed in two U.S. case–control samples comprising 100 and 98 cases, respectively (Gallagher et al. 2010). Data on premenopausal mammographic density, a strong marker of breast cancer risk, suggest a positive association with U-Cd (Adams et al. 2011), lending support to the association between Cd and breast cancer risk.

In conclusion, Cd is carcinogenic, and some but not all recent data suggest an association with certain cancer forms, even at the low dietary Cd exposures encountered in the general population. The association is present whether smokers are included or only never-smokers are studied. It appears that lung cancer and estrogen-dependent cancers are of particular importance.

## Other Effects

Cd is suspected to cause several other adverse health effects in humans, also at exposure levels found in the general population; however, results have not been consistent or causality had not been definitely demonstrated. Examples include neurodevelopmental effects (Cao et al. 2009; Ciesielski et al. 2012; Kippler et al. 2012a, 2012b), diabetes (Afridi et al. 2008; Barregard et al. 2013; Schwartz et al. 2003), and cardiovascular disease or mortality (Agarwal et al. 2011; Bao et al. 2009; Fagerberg et al. 2012, 2013; Li et al. 2011; Menke et al. 2009; Messner et al. 2009; Nakagawa et al. 2006; Nawrot et al. 2008; Nishijo et al. 2006; Peters et al. 2010; Tellez-Plaza et al. 2012b, 2013).

## Discussion

Tubular proteinuria is a well-established adverse effect associated with Cd exposure at U-Cd > 4 μg/g cr and/or B-Cd > 4 μg/L in occupationally as well as environmentally exposed populations. Cd-induced proteinuria has been associated with increased mortality in renal and cardiovascular diseases. However, in recent years, a considerable number of publications have presented evidence of an association between increased urinary excretion of proteins and the much lower U-Cd concentrations found in general populations. However, the apparent dose–response relationship for proteinuria at these low U-Cd concentrations may be noncausal (Akerström et al. 2013b; Haddam et al. 2011). Evidence of risk of chronic kidney disease (i.e., ESRD) at low exposures is very limited.

Associations with bone effects, including a decrease of bone mineral density and increased risk of osteoporotic fractures, seem to occur at low Cd exposures. In the case of bone effects, associations based on U-Cd are more conclusive than in the case of proteinuria, at least in studies of never-smokers. Moreover, low-level dietary Cd exposure (about 15 μg/day; as assessed by dietary questionnaires) has been associated with bone effects, further supporting a causal relationship between low-level exposure and adverse effects on bone. Bone effects are also of greater public health concern than increased urinary protein excretion.

The available information shows that associations with bone effects occur in population strata with low dietary Cd intake, corresponding to U-Cd as low as 0.5–2 μg/g cr. Such exposure is greatly exceeded in large populations in many parts of the world and is present even in the areas with the lowest exposure range, such as the United States (Tellez-Plaza et al. 2012a) and Europe (Pawlas et al. 2013). A formal risk assessment based on bone effects is relevant and feasible, but out of the scope of this commentary. However it is obvious that the more serious nature of bone effects compared with a slight tubular proteinuria should be considered in the health risk assessment. This could result in a much lower tolerable intake—lower than the current U.S. EPA (1 μg/kg BW and day; U.S. EPA 2014b) and Joint FAO/WHO Expert Committee on Food Additives (25 μg/kg BW and month; WHO 2011) recommendations and possibly lower than the EFSA recommendations (2.5 μg/kg BW and week; EFSA 2009a).

Cd is classified as a human carcinogen, and recent data have shown associations between low-level environmental Cd exposure and several forms of human tumors, including lung, kidney, bladder, endometrial, and breast cancer. For such common cancers, even a slight increase of risk may carry a considerable public health burden.

Therefore, based on the available information, we suggest that Cd health risk assessments for the general population should consider effects other than proteinuria. Adverse effects on bone apparently occur at lower exposures than kidney effects (U-Cd 0.5–2 vs. > 4 μg/g cr, respectively). The effects are also more important for public health. Although the available information on risk is more limited than for proteinuria, it is still sufficient for a meaningful risk assessment. The data described above strongly indicate that estimates of the risks of bone effects in never-smoking, elderly women at present constitute the most substantial information on which estimates of exposure–response considerations may be based. At the same time, for future risk assessments, more information on other non-renal effects (including cancer) is needed, with reliable data on low-level dietary Cd exposure and/or body burden.

Agricultural soils are widely contaminated with Cd to such a degree that vegetable crops accumulate the element in concentrations sufficiently high to be a threat to public health. This exposure has not decreased over the last few decades, at least not in women (Wennberg et al. 2006). The situation is thus quite different from exposure to lead (Strömberg et al. 2008; Wennberg et al. 2006) or mercury (Wennberg et al. 2006). Balance studies of Cd in topsoils indicate that the input usually exceeds removal, at least in Europe (WHO 2007). Removal is very slow, and therefore any addition of Cd has long-lasting consequences, making it important to strictly reduce any further addition of Cd. Cd input to agricultural soil mainly originates from Cd-containing phosphate fertilizers and industrial emissions, the latter resulting in long-range trans-boundary transport with deposition far from the source (WHO 2007).

## Conclusion

Current information urges a shift in the strategy for assessment of Cd risks in the general population, moving away from a unique focus on renal tubular proteinuria. Bone effects will likely contribute more than kidney effects to the overall risk. Bone effects, along with other non-renal effects such as cancer, should also be considered in the health risk assessment of Cd.

## Supplemental Material

(139 KB) PDF
