# Supplementary material for: Non-Renal Effects and the Risk Assessment of Environmental Cadmium Exposure
Source: Environ Health Perspect. 2014 Feb 25;122(5):431–8. doi: 10.1289/ehp.1307110 (PMC4014752; doi:10.1289/ehp.1307110)
Supplement: (139 KB) PDF [file ehp.1307110.s001.pdf]

Environmental Health Perspectives doi:10.1289/ehp.1307110

Supplemental Material

Non-Renal Effects and the Risk Assessment of Environmental Cadmium Exposure

Agneta Åkesson, Lars Barregard, Ingvar A. Bergdahl, Gunnar F. Nordberg, Monica Nordberg, and Staffan Skerfving

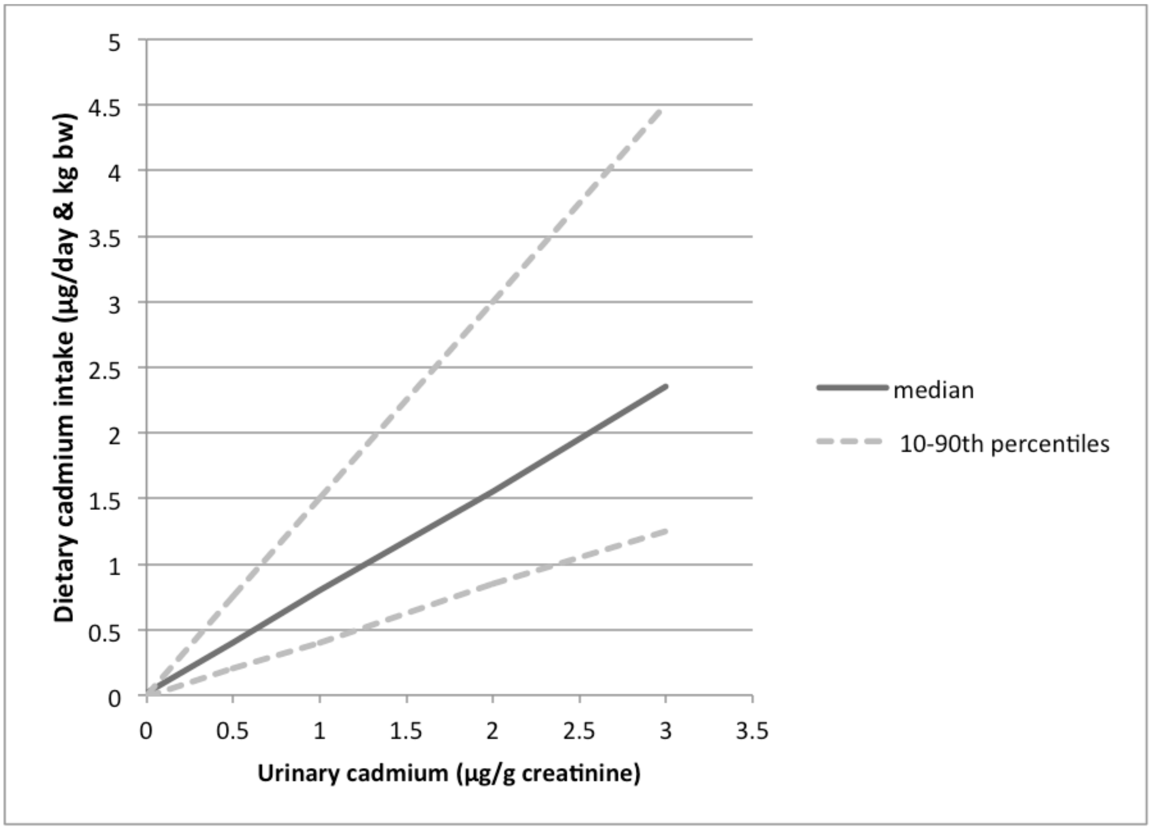

Figure S1. Modeled distribution of long-term dietary cadmium exposure corresponding to urinary cadmium concentrations at age 50 in never-smoking women (based on data from Amzal et al. 2009).

Reference

Amzal B, Julin B, Vahter M, Wolk A, Johanson G, Akesson A. 2009. Population toxicokinetic modeling of cadmium for health risk assessment. Environ Health Perspect 117:1293-1301
